# Life-course BMI trajectories and long-term weight gain in relation to hepatic steatosis in a rural community-based cohort in Southwest China

**DOI:** 10.3389/fpubh.2026.1824255

**Published:** 2026-06-18

**Authors:** Xian Cui, Jinjie Xu, Jian Wang, Lanfang Xia, Yun Fan, Liying Wang, Simei Guo, Deli Wang, Qiuyan Li, Shan Wu, Qiuyu Chen, Yao Ma, Yan Zhou, Feng Dao, Yan Su, Haiqin Wang, Jing Shi, Chunbei Yi, Qian Wang, Jun Du

**Affiliations:** 1 Diagnostic Imaging Center, Shanghai Children’s Medical Center, Shanghai Jiao Tong University School of Medicine, Shanghai, China; 2 Department of Ultrasound Medicine, Ning’er County People’s Hospital, Ning’er Hani and Yi Autonomous County, Puer, Yunnan, China; 3 Yantai Center for Disease Control and Prevention, Yantai, Shandong, China

**Keywords:** BMI trajectory, body mass index, controlled attenuation parameter, hepatic steatosis, metabolic biomarkers, restricted cubic spline, Rural China, severe steatosis

## Abstract

**Background:**

Rural communities in Southwest China are undergoing rapid nutrition and lifestyle transitions, with sustained positive energy balance and long-term weight gain. These settings are often ethnic minority-dominant and characterized by higher habitual alcohol consumption than most other Chinese populations. Evidence on how life-course adiposity relates to metabolic liver fat burden in such a rural community with varying alcohol drinking frequency remains limited. We examined associations of current BMI, life-course BMI trajectories, and long-term BMI change with controlled attenuation parameter (CAP)-defined hepatic steatosis.

**Methods:**

We conducted a retrospective cohort analysis embedded within a routine health examination program in Ning’er County, Yunnan Province, China. Adults who underwent an index examination including vibration-controlled transient elastography (VCTE) were included. Hepatic steatosis and severe steatosis were defined as CAP ≥238 and ≥292 dB/m, respectively. Body weight and height at ages 18, 30 and 40 years were extracted from prior health examination records, and BMI at each time point was calculated and categorized using Chinese cut-offs to construct life-course trajectory patterns. Multivariable logistic regression estimated odds ratios (ORs) for CAP-defined outcomes adjusting for age, sex, ethnicity, smoking, and past-year alcohol drinking frequency, with further adjustment for waist circumference and metabolic and liver biomarkers. Restricted cubic splines evaluated dose–response associations between long-term BMI change (ΔBMI from age 18 to the index examination) and steatosis.

**Results:**

Among 956 adults (predominantly ethnic minorities), the prevalence of CAP-defined steatosis and severe steatosis was 52.9 and 18.2%, respectively. Higher current BMI and weight-gain or persistent-obesity trajectory patterns were associated with greater odds of steatosis and severe steatosis. After additional adjustment, BMI ≥ 28.0 kg/m^2^ remained independently associated with steatosis (OR 2.08; 95% CI 1.05–4.14), whereas associations with severe steatosis were attenuated. Long-term BMI gain was monotonically associated with steatosis, with the OR becoming statistically significant at ΔBMI 6.64 kg/m^2^. Alcohol drinking frequency showed no strong independent association with CAP-defined outcomes.

**Conclusion:**

In this ethnic minority-dominant rural population with relatively frequent self-reported alcohol use, sustained weight gain across adulthood and adverse life-course BMI trajectories were associated with a higher burden of CAP-defined hepatic steatosis and severe steatosis. These findings support the importance of life-course weight maintenance for reducing liver fat accumulation in rural community settings.

## Introduction

Metabolic dysfunction-associated steatotic liver disease (MASLD) is now among the most prevalent chronic liver conditions worldwide and is increasingly recognized as a major contributor to cardiometabolic morbidity (1–3). In China, rapid socioeconomic transition has coincided with substantial increases in overweight and obesity, extending beyond major cities to less-resourced and rural communities where preventive services and early risk stratification are often limited (4, 5). Because liver biopsy is not feasible for population-level screening, vibration-controlled transient elastography (VCTE)-derived controlled attenuation parameter (CAP) provides a scalable, non-invasive means to quantify hepatic steatosis and identify individuals at elevated risk in community settings (6, 7).

Excess adiposity is a key driver of hepatic steatosis; however, a single current body mass index (BMI) measurement may not adequately reflect cumulative exposure to excess weight (1). Accumulating evidence supports a life-course perspective in which the timing and persistence of weight gain, such as transition to obesity in later adulthood or long-term obesity, may convey higher risk than contemporaneous BMI alone (8). Trajectory-based assessment may therefore better capture cumulative metabolic burden and inform intervention windows, yet data linking BMI trajectories to CAP-defined steatosis remain limited in community-based populations from Southwest China, where lifestyle and cardiometabolic profiles may differ from those in urban cohorts (10, 11).

Alcohol consumption may also influence hepatic steatosis and liver injury and frequently co-occurs with adverse metabolic risk factors (12). Whether alcohol drinking patterns contribute independently to CAP-defined steatosis or modify the adiposity-steatosis association in community populations remains uncertain.

In this retrospective cohort analysis embedded within a routine health examination program, we quantified hepatic steatosis using CAP measured by VCTE at the index examination in a community-based adult population. Using prior health examination records, we reconstructed BMI at ages 18, 30, and 40 years and characterized life-course BMI trajectory patterns across adulthood. We aimed to characterize the distribution and severity of CAP-defined hepatic steatosis and to examine associations of current BMI and life-course BMI trajectory patterns (ages 18, 30, and 40 years to the index examination) with CAP-defined hepatic steatosis (CAP ≥238 dB/m) and CAP-defined hepatic severe steatosis (CAP ≥292 dB/m) (13, 14). As secondary analyses, we assessed past-year alcohol drinking frequency and explored potential effect modification by adiposity. We hypothesized that trajectories involving obesity development or persistence, particularly in later adulthood, would be most strongly associated with CAP-defined steatosis.

## Methods

### Study design and participants

We conducted a retrospective cohort analysis (historical cohort) embedded within a routine health examination program in Ning’er County, Yunnan Province, Southwest China. The analysis reconstructed longitudinal adiposity exposures using anthropometric measurements recorded at prior health examinations (target ages 18, 30 and 40 years) and assessed hepatic steatosis at a single index examination using vibration-controlled transient elastography (VCTE). This study is reported in accordance with the STROBE guidelines.

### Ethics approval and consent to participate

The study protocol was reviewed and approved by the Ethics Committee of People’s Hospital of Ning’er Hani and Yi Autonomous County (approval number: PNHYIRB-P20250620-2). Written informed consent was obtained from all participants prior to data collection at the index examination. All data were de-identified before analysis.

### Participants, participant selection, and analytic samples

Adults participating in the health examination program who completed standardized questionnaires, underwent anthropometric and laboratory assessments, and received VCTE at the index examination were eligible. For the present analysis, we included participants with valid controlled attenuation parameter (CAP) measurements at the index examination. Participants with missing or invalid CA*p* values were excluded from analyses involving CAP-defined outcomes.

**Figure 1:**
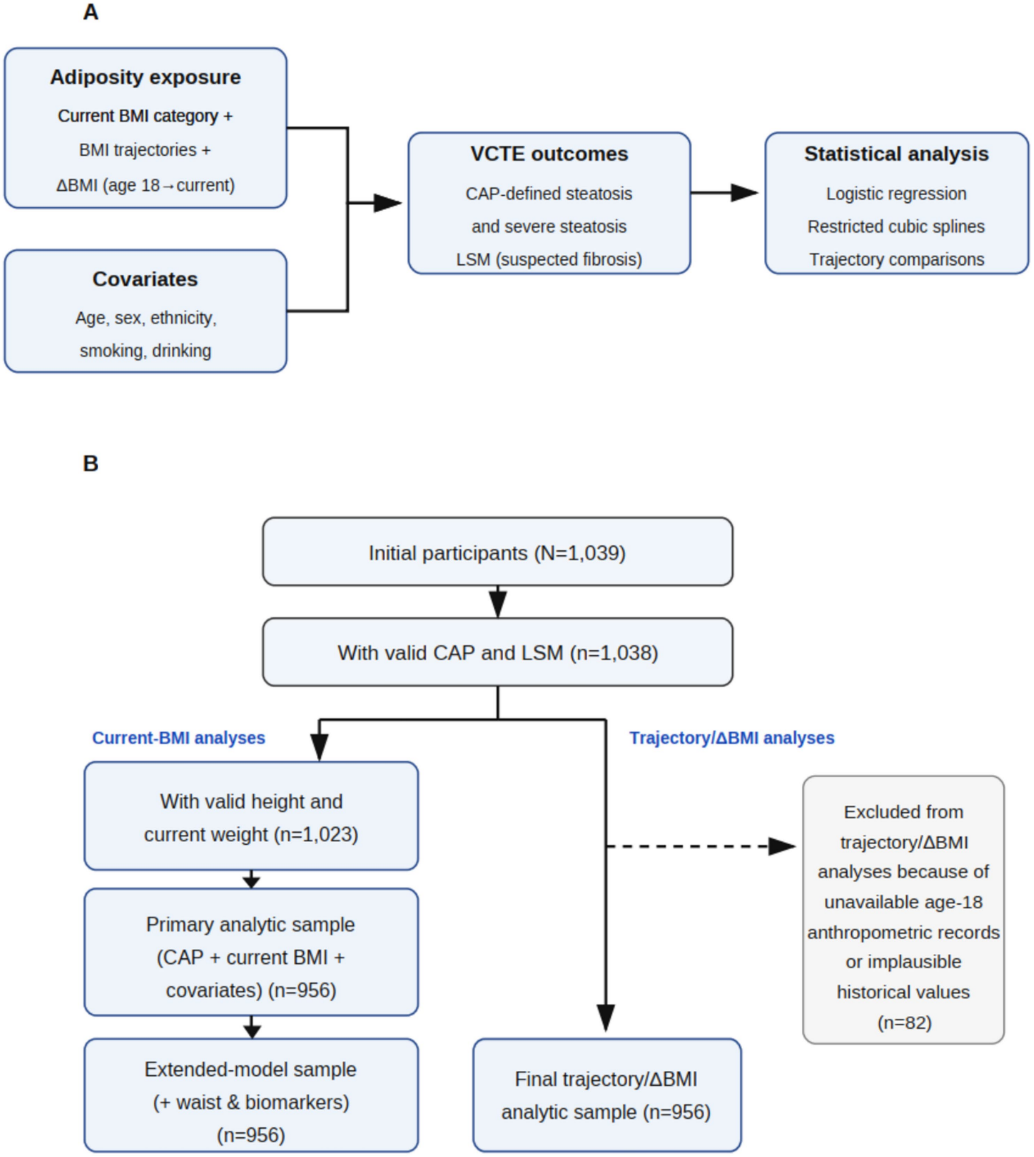
Study framework and participant flow.

The participant selection process and analytic samples are summarized in Figure 1. Of 1,039 enrolled adults, 1,038 had valid CAP measurements, and participants with missing or invalid CAP were excluded. The primary analytic sample for CAP-defined outcomes included 956 participants with available current BMI and core covariates at the index examination.

For analyses involving life-course BMI trajectories and long-term BMI change (ΔBMI), we further restricted the analytic sample to participants with valid anthropometric records at age 18 extracted from prior routine health examination records. Individuals without an age-18 record, with missing height/weight at age 18, or with implausible historical anthropometric values due to apparent weight/BMI data-entry errors (*n* = 2) were excluded from trajectory construction and ΔBMI analyses.

### Historical anthropometric data and exposure assessment

Historical height and weight at ages 18, 30 and 40 years were extracted from prior routine health examination records (rather than self-report). When multiple records were available around a target age, the measurement closest to the target age within a predefined window was selected [age window: (e.g., ±2 years)]. BMI at each time point was calculated as weight (kg)/height (m^2^). At each time point, BMI was categorized using Chinese cut-offs: <24.0 kg/m^2^ (normal), 24.0–27.9 kg/m^2^ (overweight), and ≥28.0 kg/m^2^ (obesity) (15).

Following established life-course approaches, we evaluated BMI trajectory patterns across four intervals: age 18 → 30 years, age 30 → 40 years, age 40 → current, and age 18 → current. For each interval, participants were classified into one of five trajectory patterns based on BMI categories at the two time points (16): (1) stable normal weight (normal at both time points), (2) maximum overweight (overweight at either time point but not obese at both), (3) non-obese to obese (non-obese at the earlier time point and obese at the later time point), (4) obese to non-obese (obese at the earlier time point and non-obese at the later time point), and (5) stable obesity (obese at both time points). Each interval was analyzed in separate models to minimize collinearity among highly correlated BMI measures.

### Ascertainment of hepatic steatosis and liver stiffness

Hepatic steatosis was assessed using CAP measured by VCTE (FibroScan). CAP was analyzed as a continuous measure and as categorical outcomes. Participants were categorized into four steatosis severity groups using predefined CAP thresholds: <238 dB/m, 238–258 dB/m, 259–291 dB/m, and ≥292 dB/m. For binary outcomes, steatosis was defined as CAP ≥238 dB/m and severe steatosis as CAP ≥292 dB/m. Liver stiffness measurement (LSM) was recorded concurrently as an additional liver metric. As a supplementary analysis, elevated liver stiffness was defined as LSM ≥ 8.0 kPa and was used as an additional outcome to describe the burden of suspected fibrosis in the study population.

### Alcohol drinking frequency and other covariates

Alcohol drinking frequency during the past 12 months was collected by questionnaire and operationalized as a five-level variable: 0, 1, 2, 3, or ≥4 times per week. Because alcohol exposure was assessed as drinking frequency rather than quantitative ethanol intake, we were unable to classify participants according to formal alcohol-consumption-based steatotic liver disease subtypes such as MetALD. Demographic and lifestyle covariates included age, sex, ethnicity (collapsed as Han vs. ethnic minority), and smoking status. Smoking status was defined as ever smoking, based on self-report of having smoked at least 100 cigarettes in one’s lifetime. Clinical covariates included waist circumference and routinely collected laboratory indices, including alanine aminotransferase (ALT), aspartate aminotransferase (AST), alkaline phosphatase (ALP), high-density lipoprotein cholesterol (HDL-C), triglycerides (TG), low-density lipoprotein cholesterol (LDL-C), total cholesterol (TC), fasting blood glucose (FBG), and uric acid (UA), depending on model specification.

### Statistical analysis

Participant characteristics at the index examination were summarized across CAP severity categories (<238, 238–258, 259–291, and ≥292 dB/m). Continuous variables were reported as mean (standard deviation) or median (interquartile range), and categorical variables as number (percentage), as appropriate. Continuous associations between BMI measures and CAP were assessed using Pearson correlation coefficients and visualized using scatter plots.

Multivariable logistic regression models were used to estimate odds ratios (ORs) and 95% confidence intervals (CIs) for associations of (i) current BMI category and (ii) BMI trajectory patterns with CAP-defined outcomes. Steatosis was defined as CAP ≥238 dB/m and severe steatosis as CAP ≥292 dB/m. Current BMI was categorized using Chinese cut-offs (<24.0, 24.0–27.9, and ≥28.0 kg/m^2^). BMI trajectory patterns were defined for each interval based on transitions across these BMI categories, including stable normal weight (normal at both time points), maximum overweight (overweight at either time point but not obese at both), transition from non-obese to obese, transition from obese to non-obese, and stable obesity, with stable normal weight as the reference group. Each interval was analyzed in separate models to minimize collinearity among highly correlated BMI measures.

We prespecified two sequential adjustment sets. Model 1 adjusted for age, sex, ethnicity (Han vs. ethnic minority), smoking status, and past-year alcohol drinking frequency (0/1/2/3/≥4 times per week). Model 2 additionally adjusted for waist circumference and metabolic/liver biomarkers. This model was intended as an attenuation or explanatory model rather than a primary causal confounding-adjusted model, because waist circumference and several biomarkers may lie on the pathway linking BMI to CAP-defined hepatic steatosis.

To characterize dose–response relations for long-term BMI change across adulthood, we modeled ΔBMI from age 18 to the index examination as a continuous exposure using restricted cubic splines within Model 2. The reference was set at ΔBMI = 0 kg/m^2^. From the spline-based curves, we identified clinically interpretable thresholds where the OR first became statistically significant (i.e., lower 95% CI > 1) and where the OR reached 1.5 and 2.0, respectively.

Missing values in covariates (including alcohol drinking frequency and biochemical markers) were handled using multiple imputation by chained equations (MICE) under a missing-at-random assumption. Variables included in the regression models (outcomes, exposures, and covariates), together with auxiliary predictors of missingness, were included in the imputation procedure. We generated 20 imputed datasets, fitted models within each dataset, and combined estimates using Rubin’s rules. Subgroup analyses (Model 2 only) were conducted by stratifying on prespecified factors (e.g., sex and age group), and multiplicative interaction was tested by adding an exposure×subgroup cross-product term; the *p* value for the interaction term was reported.

All tests were two-sided, and statistical significance was defined as *p* < 0.05. Analyses were performed using Python with standard statistical packages.

## Results

### Participant characteristics

Characteristics at the index examination stratified by CAP categories are shown in Table 1. Among 956 participants in the primary analytic sample, 450 (47.1%) had CAP <238 dB/m, 141 (14.8%) had CAP 238–258 dB/m, 191 (20.0%) had CAP 259–291 dB/m, and 174 (18.2%) had CAP ≥292 dB/m. Overall, 506 participants (52.9%) met the definition of hepatic steatosis (CAP ≥238 dB/m), and 174 (18.2%) met the definition of severe steatosis (CAP ≥292 dB/m).

**Table 1:** Characteristics of the study population by CAP-defined steatosis severity.

Characteristic	CAP <238 (*n* = 450)	CAP 238–258 (*n* = 141)	CAP 259–291 (*n* = 191)	CAP ≥292 (*n* = 174)	*p* value
Age (years, mean ± SD)	51.49 ± 16.51	47.80 ± 15.35	49.03 ± 15.56	46.37 ± 14.63	0.0014
Gender (*N*/%)					0.0046
Men	243 (54.00)	87 (61.70)	119 (62.30)	120 (68.97)	
Women	207 (46.00)	54 (38.30)	72 (37.70)	54 (31.03)	
Ethnicity (*N*/%)					0.0043
Han	109 (24.22)	37 (26.24)	57 (29.84)	67 (38.51)	
Minority	341 (75.78)	104 (73.76)	134 (70.16)	107 (61.49)	
Ever smoking (≥100 cigarettes in lifetime) (*N*/%)					0.2359
Yes	192 (42.67)	62 (43.97)	87 (45.55)	90 (51.72)	
No	258 (57.33)	79 (56.03)	104 (54.45)	84 (48.28)	
Past-year alcohol drinking frequency (times/week) (*N*/%)					0.0018
0	197 (43.78)	58 (41.13)	59 (30.89)	56 (32.18)	
1	76 (16.89)	10 (7.09)	33 (17.28)	21 (12.07)	
2	67 (14.89)	32 (22.70)	36 (18.85)	43 (24.71)	
3	24 (5.33)	11 (7.80)	19 (9.95)	12 (6.90)	
≥4	86 (19.11)	30 (21.28)	44 (23.04)	42 (24.14)	
Waist circumference (cm, mean ± SD)	79.93 ± 9.24	88.42 ± 8.39	90.80 ± 8.17	95.31 ± 10.52	<0.001
Total cholesterol (mmol/L, mean ± SD)	4.89 ± 1.06	5.23 ± 1.17	5.24 ± 1.45	5.59 ± 1.46	<0.001
Triglycerides (mmol/L, mean ± SD)	1.66 ± 1.16	2.52 ± 2.17	2.89 ± 2.73	4.21 ± 5.12	<0.001
HDL-C (mmol/L, mean ± SD)	1.41 ± 0.39	1.20 ± 0.33	1.21 ± 0.40	1.13 ± 0.37	<0.001
LDL-C (mmol/L, mean ± SD)	2.81 ± 0.83	3.13 ± 0.86	3.06 ± 0.97	3.23 ± 1.00	<0.001
ALT (IU/L, mean ± SD)	25.01 ± 23.28	29.64 ± 23.39	33.71 ± 23.22	45.25 ± 36.18	<0.001
AST (IU/L, mean ± SD)	25.32 ± 21.90	25.79 ± 20.49	31.28 ± 31.91	33.76 ± 39.58	0.0025
ALP (IU/L, mean ± SD)	85.37 ± 33.94	84.05 ± 36.54	87.81 ± 28.03	87.13 ± 23.52	0.6915
Blood glucose (mmol/L, mean ± SD)	5.37 ± 1.97	5.56 ± 1.81	5.63 ± 1.48	5.91 ± 1.74	0.0177
HbA1c (%, mean ± SD)	7.57 ± 2.90	7.20 ± 2.48	6.47 ± 1.59	7.04 ± 1.55	0.7960
Uric acid (μmol/L, mean ± SD)	349.52 ± 114.58	369.62 ± 101.70	410.62 ± 112.35	417.78 ± 114.17	<0.001
BMI at age 18 (kg/m^2^, mean ± SD)	20.76 ± 3.09	21.65 ± 3.77	21.97 ± 3.85	21.75 ± 3.87	<0.001
BMI at age 30 (kg/m^2^, mean ± SD)	21.95 ± 3.45	23.28 ± 4.02	23.44 ± 3.98	23.75 ± 4.22	<0.001
BMI at age 40 (kg/m^2^, mean ± SD)	22.78 ± 3.33	24.27 ± 4.06	24.15 ± 3.85	24.52 ± 4.47	<0.001
Current BMI (kg/m^2^, mean ± SD)	22.48 ± 3.42	25.15 ± 3.24	26.64 ± 3.23	28.00 ± 4.02	<0.001
Median CAP (dB/m, mean ± SD)	203.34 ± 25.09	248.66 ± 5.79	273.21 ± 9.59	320.25 ± 21.08	<0.001
LSM (kPa, mean ± SD)	5.16 ± 4.50	5.07 ± 3.49	6.19 ± 6.72	6.28 ± 3.08	0.0066

CAP, controlled attenuation parameter; LSM, liver stiffness measurement; ALT, alanine aminotransferase; AST, aspartate aminotransferase; ALP, alkaline phosphatase; HDL-C, high-density lipoprotein cholesterol; LDL-C, low-density lipoprotein cholesterol; TC, total cholesterol; TG, triglycerides. CAP categories were defined as CAP <238 dB/m, 238–258 dB/m, 259–291 dB/m, and ≥292 dB/m.

Mean age was broadly comparable across CAP categories (51.49 ± 16.51 years for CAP <238; 47.80 ± 15.35 for CAP 238–258; 49.03 ± 15.56 for CAP 259–291; and 46.37 ± 14.63 for CAP ≥292; Table 1). The proportion of men increased with steatosis severity (54.0% in CAP <238 vs. 69.0% in CAP ≥292). Smoking prevalence showed a modest increasing trend (42.7 to 51.7%), and the proportion reporting frequent drinking (≥4 times/week) was slightly higher in more severe CAP groups (19.1 to 24.1%).

Across increasing CAP categories, adiposity measures and metabolic biomarkers showed a graded pattern. Waist circumference and BMI at each adulthood time point and at the current examination increased monotonically with steatosis severity. Higher CAP categories were also characterized by higher ALT/AST, triglycerides, LDL-C and uric acid levels and lower HDL-C (Table 1).

### Current adiposity and CAP-defined steatosis

**Figure 2:**
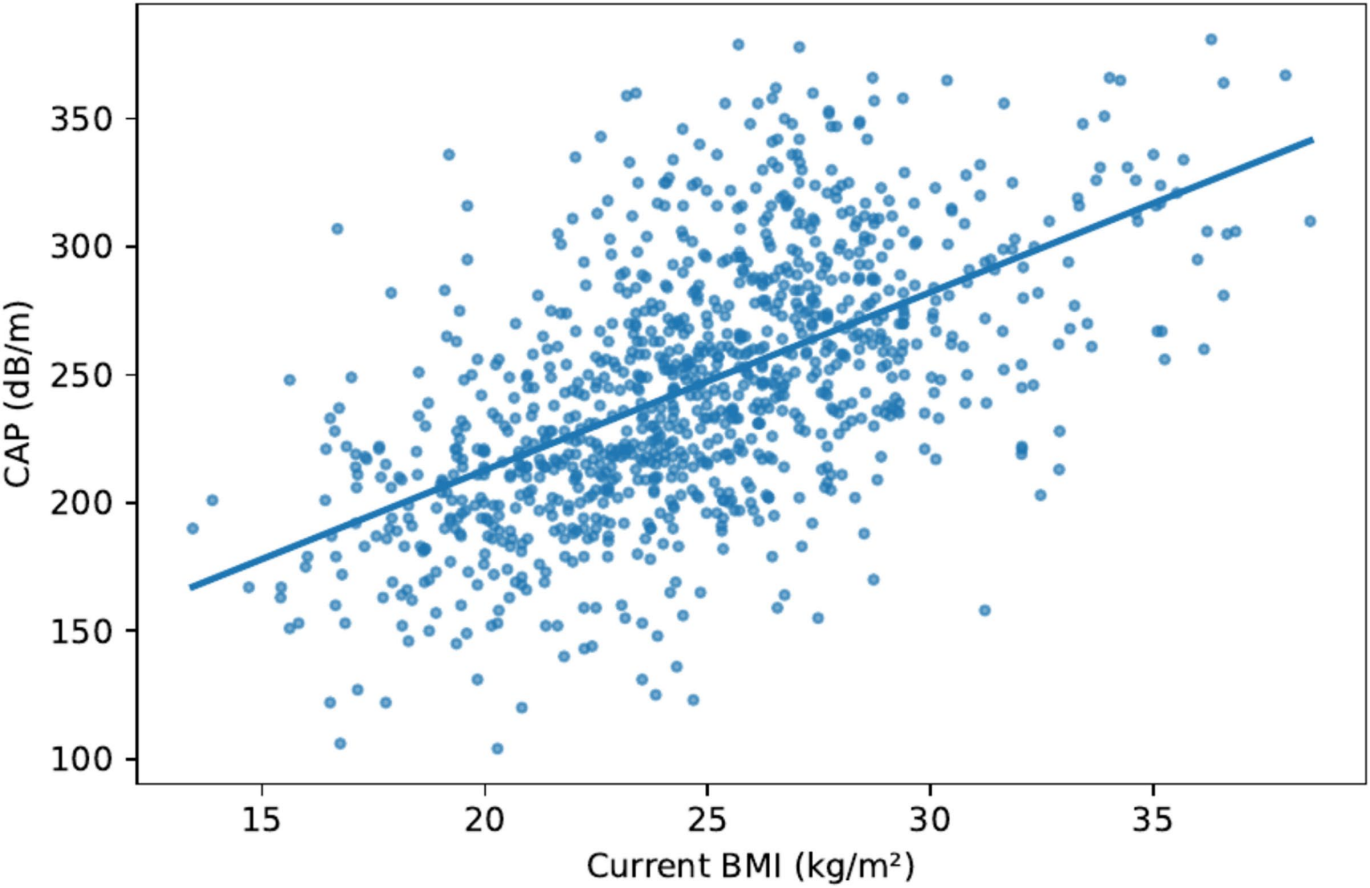
Association between current BMI and CAP.

Figure 2 illustrates a clear positive association between current BMI and CAP as continuous measures. In multivariable logistic regression models (Table 2, Model 1), current BMI category showed a strong dose–response relationship with steatosis (CAP ≥238 dB/m). Compared with BMI < 24 kg/m^2^, the adjusted ORs were 5.54 (95% CI 4.03–7.62) for BMI 24–27.9 kg/m^2^ and 18.36 (95% CI 11.37–29.66) for BMI ≥ 28 kg/m^2^. Associations were similar for severe steatosis (CAP ≥292 dB/m; Table 2, Model 1), with ORs of 4.84 (2.92–8.01) for BMI 24–27.9 kg/m^2^ and 11.46 (6.74–19.49) for BMI ≥ 28 kg/m^2^.

**Table 2:** Associations of current BMI categories with CAP-defined steatosis and severe steatosis.

Exposure	CAP ≥238 (OR, 95%CI)		CAP ≥292 (OR, 95%CI)	
	Model 1^a^	Model 2^b^	Model 1^a^	Model 2^b^
BMI < 24 kg/m^2^	Reference	Reference	Reference	Reference
BMI 24–27.9 kg/m^2^	5.59 (4.06–7.69)	1.64 (1.06–2.53)	4.86 (2.94–8.04)	1.49 (0.81–2.76)
BMI ≥ 28 kg/m^2^	18.37 (11.37–29.67)	2.08 (1.05–4.14)	11.46 (6.74–19.50)	1.33 (0.59–3.03)

^a^Model 1 adjusted for age, sex, ethnicity, smoking status, and past-year alcohol drinking frequency. ^b^Model 2 additionally adjusted for waist circumference and metabolic/liver biomarkers (ALT, AST, ALP, HDL-C, LDL-C, triglycerides, total cholesterol, fasting blood glucose, uric acid). CAP, controlled attenuation parameter; OR: odds ratios; CI, confidence intervals.

After additional adjustment for waist circumference and biochemical markers (Table 2, Model 2), the associations for BMI categories were substantially attenuated. For CAP ≥238 dB/m, the ORs were 1.64 (1.06–2.53) for BMI 24–27.9 kg/m^2^ and 2.08 (1.05–4.14) for BMI ≥ 28 kg/m^2^. For CAP ≥292 dB/m, neither BMI category remained independently associated [BMI 24–27.9: OR 1.49 (0.81–2.76); BMI ≥ 28: OR 1.33 (0.59–3.03)], whereas waist circumference and selected metabolic markers retained robust associations (Table 2).

Scatter plot showing the association between current Body Mass Index (BMI, kg/m2) and Controlled Attenuation Parameter (CAP). The solid blue line represents the linear regression trend, indicating a positive correlation between adiposity and hepatic steatosis. Each blue dot represents an individual participant.

### Dose–response and threshold patterns for ΔBMI (18 → current)

**Figure 3:**
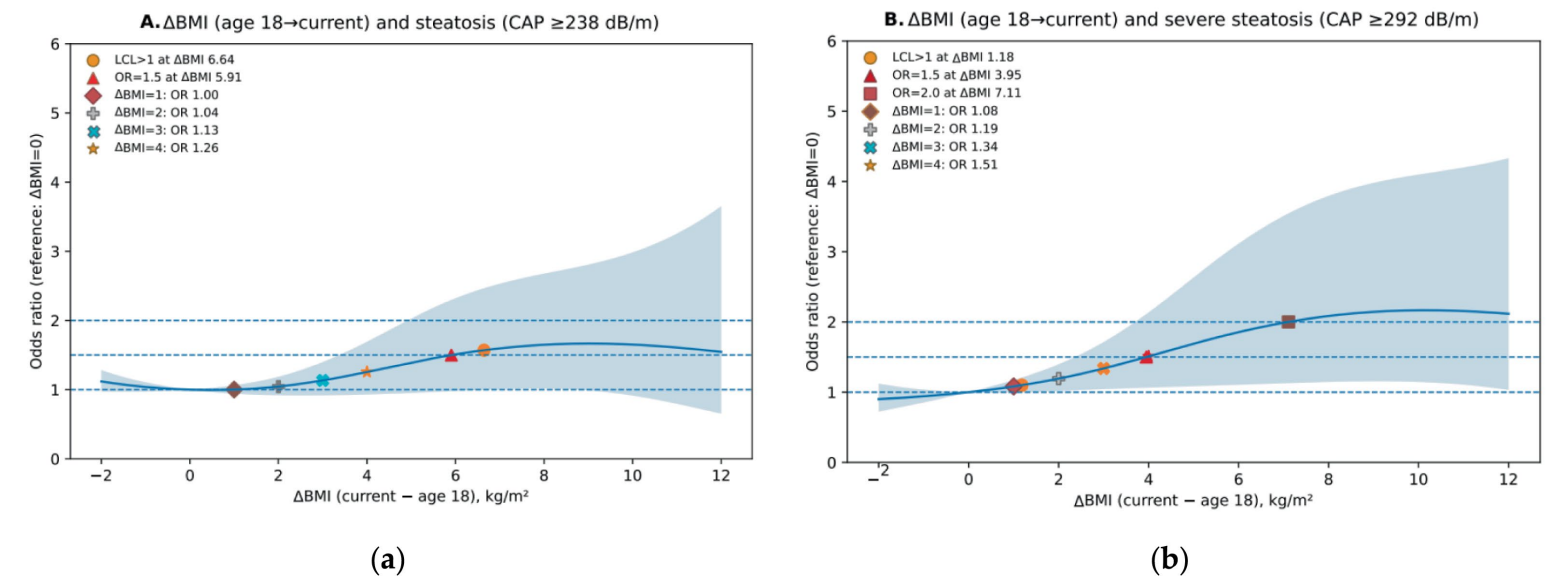
Risk of steatosis according to BMI changes from early adulthood. Odds ratios (OR) and 95% confidence intervals for **(a)** steatosis (CAP ≥238 dB/m) and **(b)** severe steatosis (CAP ≥ 292 dB/m) associated with BMI change (ΔBMI) from age 18 to current age.

In spline-based Model 2 analyses, ΔBMI from age 18 to the index examination was monotonically associated with higher odds of CAP-defined outcomes (Figure 3). For steatosis (CAP ≥238 dB/m), the OR reached 1.5 at ΔBMI = 5.91 kg/m^2^ and became statistically significant (lower 95% CI > 1) at ΔBMI = 6.64 kg/m^2^. For severe steatosis (CAP ≥292 dB/m), the lower CI first exceeded 1 at ΔBMI = 1.18 kg/m^2^; the OR reached 1.5 at ΔBMI = 3.95 kg/m^2^ and 2.0 at ΔBMI = 7.11 kg/m^2^.

### BMI trajectory patterns and CAP-defined steatosis

**Figure 4:**
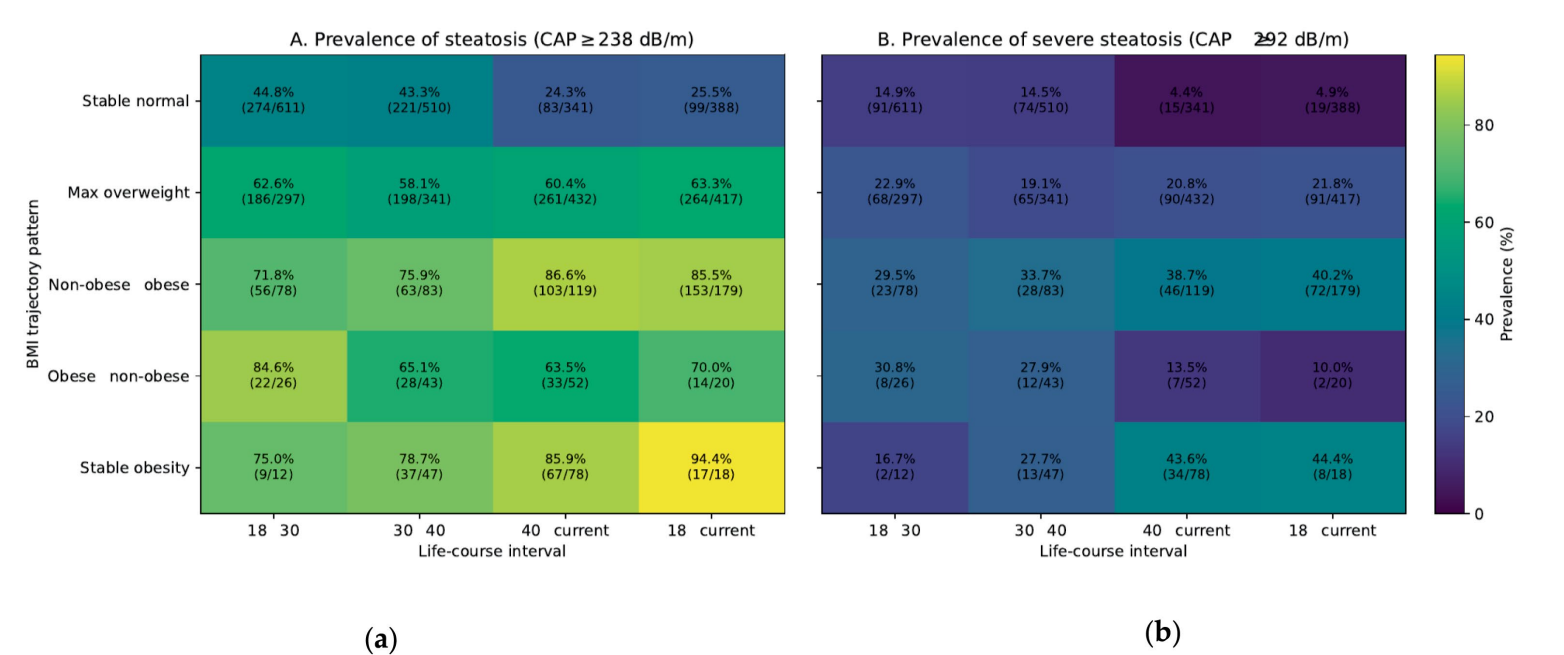
Prevalence of CAP-defined outcomes across BMI trajectory patterns. Heatmap of the prevalence of **(a)** steatosis and **(b)** severe steatosis categorized by five distinct BMI trajectory patterns across four life-course intervals.

Using BMI cut-offs of <24/24–27.9 / ≥28 kg/m^2^, the prevalence of CAP-defined steatosis differed markedly across BMI trajectory patterns and life-course intervals (Figure 4). Trajectories characterized by weight gain or persistent obesity consistently showed higher prevalence of both steatosis (CAP ≥238 dB/m) and severe steatosis (CAP ≥292 dB/m), whereas the stable normal-weight pattern showed the lowest prevalence across intervals.

In multivariable models, these patterns translated into substantially higher odds of steatosis and severe steatosis compared with stable normal weight (Table 3). Detailed sample sizes and event counts for each trajectory category are provided in Supplementary Table S1. In Model 1, trajectories closer to the current period generally showed larger effect sizes. For example, from age 40 to the index examination, compared with stable normal weight, maximum overweight was associated with an OR of 4.41 (95% CI 3.16–6.14), transition from non-obese to obese with an OR of 20.68 (11.21–38.15), and stable obesity with an OR of 19.70 (9.76–39.75). Participants transitioning from obese to non-obese also showed elevated odds (OR 5.07, 2.67–9.63). From age 18 to the index examination, the corresponding ORs were 3.76 (2.64–5.35) for maximum overweight, 19.95 (11.78–33.78) for non-obese to obese transition, 9.04 (3.26–25.08) for obese to non-obese transition, and 48.44 (6.21–378.02) for stable obesity, noting wide confidence intervals for stable obesity consistent with limited numbers in this trajectory group (Table 3). Detailed sample sizes and event counts for each trajectory category are provided in Supplementary Table S1.

**Table 3:** BMI trajectory patterns across life-course intervals and CAP-defined outcomes.

Interval (years)	Trajectory pattern (ref: Stable normal)^a^	CAP ≥238 (OR, 95%CI)		CAP ≥292 (OR, 95%CI)	
		Model 1^b^	Model 2^c^	Model 1^b^	Model 2^c^
18 → 30	Maximum overweight	2.19 (1.62–2.96)	1.35 (0.93–1.98)	1.85 (1.27–2.70)	1.18 (0.75–1.85)
18 → 30	Non-obese → obese	3.29 (1.93–5.63)	1.30 (0.65–2.60)	2.50 (1.42–4.42)	1.22 (0.62–2.39)
18 → 30	Obese → non-obese	7.54 (2.52–22.59)	5.27 (1.46–19.02)	2.46 (0.99–6.08)	1.41 (0.46–4.34)
18 → 30	Stable obesity	4.16 (1.10–15.75)	1.01 (0.19–5.52)	1.34 (0.28–6.33)	0.16 (0.02–1.57)
30 → 40	Maximum overweight	1.98 (1.47–2.67)	1.04 (0.71–1.52)	1.51 (1.02–2.24)	0.71 (0.44–1.16)
30 → 40	Non-obese → obese	4.29 (2.47–7.46)	1.13 (0.56–2.25)	3.22 (1.85–5.60)	0.93 (0.47–1.83)
30 → 40	Obese → non-obese	2.55 (1.31–4.96)	1.09 (0.45–2.66)	2.38 (1.14–4.95)	1.15 (0.48–2.75)
30 → 40	Stable obesity	5.41 (2.57–11.37)	1.08 (0.43–2.72)	2.42 (1.16–5.05)	0.55 (0.23–1.33)
40 → Current	Maximum overweight	4.43 (3.18–6.18)	1.54 (1.00–2.35)	4.95 (2.77–8.84)	1.83 (0.94–3.56)
40 → Current	Non-obese → obese	20.69 (11.21–38.17)	2.09 (0.96–4.55)	11.29 (5.84–21.83)	1.17 (0.46–2.93)
40 → Current	Obese → non-obese	5.08 (2.67–9.64)	1.57 (0.72–3.39)	2.57 (0.93–7.09)	0.67 (0.21–2.15)
40 → Current	Stable obesity	19.69 (9.76–39.75)	1.74 (0.70–4.32)	16.45 (8.11–33.34)	1.85 (0.71–4.81)
18 → Current	Maximum overweight	4.74 (3.44–6.53)	1.69 (1.12–2.53)	4.68 (2.76–7.94)	1.66 (0.89–3.08)
18 → Current	Non-obese → obese	17.84 (10.86–29.32)	2.02 (1.03–3.96)	11.84 (6.72–20.86)	1.42 (0.63–3.22)
18 → Current	Obese → non-obese	8.02 (2.93–21.93)	4.06 (1.29–12.75)	2.15 (0.45–10.18)	0.61 (0.08–4.91)
18 → Current	Stable obesity	44.48 (5.76–343.31)	3.72 (0.42–32.89)	11.23 (3.84–32.84)	1.33 (0.35–5.07)

^a^Trajectory patterns were defined using BMI cut-offs of <24/24–27.9 / ≥28 kg/m2. Reference group: stable normal weight. ^b^Model 1 adjusted for age, sex, ethnicity, smoking status, and past-year alcohol drinking frequency. ^c^Model 2 additionally adjusted for waist circumference and metabolic/liver biomarkers (ALT, AST, ALP, HDL-C, LDL-C, triglycerides, total cholesterol, fasting blood glucose, uric acid). BMI, body mass index; CAP, controlled attenuation parameter; OR: odds ratios; CI, confidence intervals.

In Model 2, associations for trajectory patterns were generally attenuated after additional adjustment for waist circumference and biochemical markers, but weight-gain and obesity-persistent patterns remained associated with higher odds of CAP ≥238 dB/m in several intervals (Table 3).

### Secondary analyses

Subgroup analyses using Model 2 showed broadly consistent trajectory-steatosis associations across sex, age group, ethnicity, and smoking status, with most *p* values for interaction not statistically significant (Supplementary Tables S2–S5). One nominal interaction was observed for severe steatosis (CAP ≥292 dB/m) in the 18 → 30 interval by smoking status, but patterns were not consistently heterogeneous across intervals (Supplementary Tables S2–S5).

Past-year drinking frequency was included as a covariate in the primary models and showed no clear independent association with CAP-defined outcomes (Table 2). In interaction analyses, no strong overall evidence of multiplicative interaction between current BMI category and alcohol drinking frequency was observed (overall interaction *p* = 0.503; Supplementary Table S6), although one individual interaction term for BMI ≥ 28 kg/m^2^ combined with alcohol frequency of 2 times/week was borderline significant (OR 5.27, 95% CI 1.00–27.73; *p* = 0.050). In a supplementary analysis, elevated liver stiffness (LSM ≥ 8.0 kPa) was present in 6.4% (61/956) of participants and increased across CAP-defined steatosis categories, from 4.7% in participants with CAP <238 dB/m to 10.9% in those with CAP ≥292 dB/m. Higher CAP-defined steatosis severity, but not current BMI category, was associated with elevated LSM in core-adjusted models (Supplementary Table S7).

## Discussion

In this community-based health examination cohort from rural Southwest China, we observed a high burden of CAP-defined hepatic steatosis and severe steatosis. Across multiple adiposity metrics, current BMI, life-course BMI trajectory patterns, and long-term BMI change from early adulthood were consistently associated with higher odds and prevalence of steatosis. Notably, weight-gain and persistent-obesity trajectories showed the most adverse risk profiles, while the stable normal-weight pattern remained the lowest-risk group across intervals. These findings underscore that how weight accumulates across adulthood, beyond a single contemporaneous BMI value, captures meaningful heterogeneity in liver fat burden in resource-limited settings.

Our trajectory findings align with longitudinal evidence that weight gain during young adulthood and across adulthood is associated with higher later-life CAP-defined hepatic steatosis prevalence, even after accounting for metabolic risk factors. For example, analyses from CARDIA demonstrated that distinct BMI trajectories in young adulthood predicted CAP-defined hepatic steatosis in midlife, highlighting the importance of weight maintenance over the adult life course (17, 18). Similarly, other cohort studies have linked weight gain since early adulthood to CAP-defined hepatic steatosis risk, supporting the concept of cumulative adiposity exposure as a key driver of liver steatosis (17, 19). Our results extend this literature by demonstrating analogous patterns using VCTE-derived CAP outcomes in a rural Chinese population, where adult weight gain is increasingly common yet prevention resources are often limited.

An important observation in our data is the elevated risk among participants transitioning from obese to non-obese categories. This may reflect “metabolic memory” or residual risk after prior obesity exposure, including persistent visceral adiposity, ectopic fat partitioning, or incomplete normalization of hepatic lipid handling despite later BMI improvement (20, 21). Although this retrospective cohort analysis with CAP assessed at the index examination cannot distinguish intentional from unintentional weight loss from illness-related weight change, prior studies suggest that lifetime exposure to higher BMI can carry lasting cardiometabolic consequences, and our results suggest that liver fat accumulation may follow a similar pattern.

Beyond trajectory categories, we leveraged restricted cubic splines to characterize the dose–response shape for ΔBMI (age 18 → the index examination). The monotonic association and the emergence of statistically significant elevation in steatosis odds at ΔBMI ≈6.6 kg/m^2^ provides an interpretable “risk escalation” point that may be useful for communication and screening prioritization (22). Importantly, this threshold should be interpreted as a population-level marker rather than a deterministic clinical cutoff: the precise value may vary with measurement error in historical anthropometric assessment, body composition differences, and metabolic context (23, 24). Nonetheless, our approach complements categorical trajectory analyses by quantifying how incremental long-term adiposity accrual translates into liver fat risk.

A central finding is the substantial attenuation of BMI associations after adjustment for waist circumference and metabolic biomarkers, with waist circumference remaining robustly associated with CAP-defined steatosis (25). This pattern supports a pathway in which general adiposity (BMI) contributes to hepatic steatosis largely through visceral/abdominal fat accumulation and downstream metabolic dysregulation. Prior work has emphasized the adipocentric nature of steatotic liver disease, where increased lipid flux from adipose tissue, enhanced hepatic *de novo* lipogenesis, and impaired lipid export collectively drive intrahepatic triglyceride accumulation (26, 27). Reviews and mechanistic studies further implicate insulin resistance as a key upstream driver of hepatic lipid overload and de novo lipogenesis, offering biologic plausibility for why metabolic covariates and waist circumference “explain” a large share of the BMI-steatosis association in multivariable models (27, 28).

The graded increases in triglycerides, LDL-C, uric acid, and liver enzymes across CAP categories in our cohort are consistent with this framework. Hyperuricemia, in particular, has been repeatedly associated with MASLD risk in meta-analyses, and may reflect shared pathways involving oxidative stress, insulin resistance, and hepatic lipid metabolism (29, 30). Although our analyses were not designed for mediation inference, the attenuation observed in Model 2 suggests that interventions targeting abdominal adiposity and metabolic health, rather than BMI alone, may be most impactful for reducing liver fat burden in similar settings. Model 2 is best interpreted as an attenuation model rather than a strict independent-effect model, because waist circumference and several metabolic biomarkers may lie on the pathway between BMI and CAP-defined steatosis. The stronger attenuation observed for CAP-defined severe steatosis likely reflects adjustment for more proximal adiposity/metabolic markers, together with reduced precision due to the smaller number of severe steatosis cases.

We used commonly applied CAP thresholds to define steatosis severity (≥238 dB/m for steatosis; ≥292 dB/m for severe steatosis) (31, 32). These cut points have been used in prior studies and clinical interpretive guidance for CAP-based staging, providing a pragmatic framework for population stratification (29, 33, 34). CAP is an established noninvasive tool for steatosis assessment, though its performance varies by BMI, device/probe selection, and underlying histologic spectrum; meta-analytic evidence supports its utility for detecting steatosis with reasonable accuracy while acknowledging heterogeneity across studies (35). In this context, our findings are best interpreted as associations with CAP-defined liver fat burden rather than biopsy-confirmed histologic MASLD/MASH, and future work incorporating standardized VCTE protocols and (where feasible) reference imaging/biopsy would strengthen causal and clinical inference.

Alcohol consumption should be interpreted cautiously in this cohort. Alcohol drinking frequency was included as a covariate in the primary models and showed no clear independent association with CAP-defined outcomes. This observation is generally consistent with the emerging steatotic liver disease framework, in which metabolic dysfunction and alcohol exposure are considered overlapping but distinct contributors to liver fat accumulation (36). However, alcohol exposure in our study was assessed using self-reported past-year drinking frequency rather than quantitative ethanol intake, drinking duration, or drinking pattern such as binge drinking; therefore, we were unable to formally classify participants according to alcohol-related steatotic liver disease subtypes such as MetALD. In interaction analyses, we found limited evidence that alcohol drinking frequency modified the association between current BMI and CAP-defined steatosis. Although one individual interaction term for BMI ≥ 28 kg/m^2^ combined with drinking twice per week was borderline significant, the confidence interval was wide and the overall interaction test was not statistically significant. Accordingly, this finding should be regarded as exploratory rather than confirmatory. Our results should therefore be interpreted as associations of current BMI and life-course weight gain with CAP-defined hepatic steatosis, rather than evidence for a clinically adjudicated MASLD or MetALD phenotype.

From a prevention standpoint, our results suggest two practical messages. First, weight maintenance across adulthood, particularly avoiding sustained upward BMI trajectories, may meaningfully reduce steatosis burden, consistent with cohort evidence emphasizing early and sustained prevention (37). Second, the attenuation after waist circumference and metabolic adjustment highlights that abdominal adiposity and metabolic risk control (lipids, glycemia, uric acid, and liver enzymes as risk signals) may be efficient targets for screening and intervention triage in rural health systems. Lifestyle interventions achieving weight loss have demonstrated improvements in liver histology and steatosis outcomes, providing an evidence base for scalable behavioral approaches (38, 39).

Key strengths include CAP-based phenotyping in a community setting, the integration of life-course BMI patterns and long-term ΔBMI modeling, and the use of spline methods to characterize dose–response relationships. Several limitations merit consideration. First, although adiposity exposures were reconstructed from historical examination records, CAP was assessed at a single index examination; therefore, causal direction cannot be confirmed and cannot separate intentional from unintentional weight change. Because the trajectory analyses required historical anthropometric records at age 18, exclusion of participants without such records may have introduced selection bias and may limit the generalizability of the life-course trajectory findings. Second, historical anthropometric data were obtained from prior routine examination records; nevertheless, records may be incomplete, measured at slightly different ages, or reflect varying measurement conditions, which may introduce exposure misclassification. Several trajectory-specific and subgroup-specific estimates were imprecise, particularly for rare categories such as stable obesity and obese-to-non-obese transitions in earlier life-course intervals. These sparse-data patterns resulted in wide confidence intervals or non-estimable cells and limited statistical power for some subgroup analyses. Third, CAP thresholds and measurement variability (including probe selection and operator factors) may lead to misclassification, although nondifferential misclassification would generally attenuate estimates (35). Fourth, although MASLD provides the contemporary conceptual framework for steatotic liver disease, our study outcomes were defined using CAP thresholds and should therefore be interpreted as CAP-defined steatosis phenotypes rather than formal MASLD diagnoses based on full metabolic criteria. In addition, alcohol exposure was measured using drinking frequency rather than quantitative ethanol intake; therefore, formal classification into MetALD or related alcohol-based categories was not feasible. Finally, while we adjusted for multiple confounders and metabolic markers, residual confounding and overadjustment (if biomarkers lie on the causal pathway) remain possible; therefore, Model 1 and Model 2 should be interpreted as complementary perspectives (core association vs. association independent of abdominal/metabolic context), rather than competing “correct” estimates.

## Conclusion

In this retrospective cohort analysis from rural Southwest China, sustained weight gain across adulthood and adverse life-course BMI trajectory patterns were associated with greater metabolic liver fat burden assessed by transient elastography at the index examination. Weight-gain and persistent-obesity trajectories consistently showed the highest odds of CAP-defined hepatic steatosis, while stable normal weight showed the lowest burden. Long-term BMI change from age 18 to the index examination displayed a monotonic dose–response relationship, with thresholds at which steatosis risk increased and became statistically significant. These findings, observed in an ethnic minority–dominant rural population with relatively frequent alcohol use, support life-course weight maintenance and prevention of sustained upward BMI trajectories as pragmatic strategies to mitigate ectopic liver fat accumulation in resource-limited communities.

## Data Availability

The original contributions presented in the study are included in the article/Supplementary material, further inquiries can be directed to the corresponding author/s.
