# Supplementary material for: Life-course BMI trajectories and long-term weight gain in relation to hepatic steatosis in a rural community-based cohort in Southwest China
Source: Front Public Health. 2026 Jun 18;14:1824255. doi: 10.3389/fpubh.2026.1824255 (PMC13323307; doi:10.3389/fpubh.2026.1824255)
Supplement: Supplementary file 1 [file Table_1.DOCX]

**Supplementary Table S1. Sample size and event counts for BMI trajectory patterns across life-course intervals**

| **Interval** | **Trajectory pattern** | **n** | **CAP ≥238 events** | **CAP ≥292 events** |
| --- | --- | --- | --- | --- |
| 18→30 | Stable normal | 551 | 240 | 75 |
| 18→30 | Maximum overweight | 295 | 184 | 67 |
| 18→30 | Non-obese → obese | 78 | 56 | 23 |
| 18→30 | Obese → non-obese | 25 | 21 | 7 |
| 18→30 | Stable obesity | 12 | 9 | 2 |
| 30→40 | Stable normal | 448 | 185 | 57 |
| 30→40 | Maximum overweight | 340 | 197 | 64 |
| 30→40 | Non-obese → obese | 83 | 63 | 28 |
| 30→40 | Obese → non-obese | 43 | 28 | 12 |
| 30→40 | Stable obesity | 47 | 37 | 13 |
| 40→Current | Stable normal | 321 | 79 | 14 |
| 40→Current | Maximum overweight | 399 | 235 | 79 |
| 40→Current | Non-obese → obese | 111 | 96 | 40 |
| 40→Current | Obese → non-obese | 52 | 33 | 7 |
| 40→Current | Stable obesity | 78 | 67 | 34 |
| 18→Current | Stable normal | 368 | 95 | 18 |
| 18→Current | Maximum overweight | 384 | 238 | 80 |
| 18→Current | Non-obese → obese | 172 | 147 | 67 |
| 18→Current | Obese → non-obese | 20 | 14 | 2 |
| 18→Current | Stable obesity | 17 | 16 | 7 |

Trajectory patterns were defined using Chinese BMI cut-offs (<24.0, 24.0–27.9, and ≥28.0 kg/m²). CAP ≥238 dB/m indicates CAP-defined hepatic steatosis; CAP ≥292 dB/m indicates CAP-defined severe steatosis. Counts were calculated from the trajectory analytic records available in the uploaded dataset. The table is intended to show cell sizes and event counts for assessing statistical precision, especially in rare trajectory categories.

**Supplementary Table S2. Subgroup analyses of BMI trajectory patterns in relation to CAP-defined steatosis and severe steatosis by sex**

| **Sex level** | **Interval** | **Trajectory pattern (ref: Stable normal)** | **CAP ≥238 OR (95% CI)** | **CAP ≥292 OR (95% CI)** | **P for interaction (CAP ≥238)** | **P for interaction (CAP ≥292)** |
| --- | --- | --- | --- | --- | --- | --- |
| Men | 18→30 | Maximum overweight | 1.58 (0.81-3.05) | 1.52 (0.76-3.06) | 0.875 | 0.294 |
|  | 18→30 | Non-obese → obese | 0.95 (0.24-3.66) | 3.65 (1.14-11.62) |  |  |
|  | 18→30 | Obese → non-obese | 1.19 (0.14-10.26) | 1.57 (0.19-12.83) |  |  |
|  | 18→30 | Stable obesity | 0.59 (0.05-6.42) | 0.28 (0.02-4.74) |  |  |
|  | 30→40 | Maximum overweight | 1.52 (0.80-2.89) | 0.74 (0.34-1.59) | 0.551 | 0.405 |
|  | 30→40 | Non-obese → obese | 2.37 (0.65-8.67) | 0.97 (0.33-2.89) |  |  |
|  | 30→40 | Obese → non-obese | 0.68 (0.16-2.85) | 1.79 (0.48-6.70) |  |  |
|  | 30→40 | Stable obesity | 1.93 (0.18-20.78) | 1.50 (0.27-8.21) |  |  |
|  | 40→Current | Maximum overweight | 1.59 (0.80-3.18) | 2.05 (0.69-6.07) | 0.572 | 0.801 |
|  | 40→Current | Non-obese → obese | 5.77 (1.05-31.71) | 1.12 (0.26-4.74) |  |  |
|  | 40→Current | Obese → non-obese | 2.33 (0.62-8.74) | 1.43 (0.25-8.16) |  |  |
|  | 40→Current | Stable obesity | NA | 2.61 (0.56-12.17) |  |  |
|  | 18→Current | Maximum overweight | 1.54 (0.80-2.95) | 2.04 (0.75-5.54) | 0.257 | 0.980 |
|  | 18→Current | Non-obese → obese | 5.61 (1.07-29.42) | 1.73 (0.49-6.12) |  |  |
|  | 18→Current | Obese → non-obese | 0.32 (0.03-4.14) | NA |  |  |
|  | 18→Current | Stable obesity | NA | 1.32 (0.18-9.72) |  |  |
| Women | 18→30 | Maximum overweight | 0.96 (0.50-1.85) | 0.79 (0.34-1.86) | 0.875 | 0.294 |
|  | 18→30 | Non-obese → obese | 0.84 (0.24-2.92) | 0.60 (0.16-2.22) |  |  |
|  | 18→30 | Obese → non-obese | 1.73 (0.16-18.25) | NA |  |  |
|  | 18→30 | Stable obesity | 0.53 (0.04-7.80) | 0.05 (0.00-4.15) |  |  |
|  | 30→40 | Maximum overweight | 0.89 (0.46-1.72) | 0.64 (0.26-1.59) | 0.551 | 0.405 |
|  | 30→40 | Non-obese → obese | 0.90 (0.29-2.77) | 0.55 (0.15-1.97) |  |  |
|  | 30→40 | Obese → non-obese | 1.08 (0.20-5.92) | 1.62 (0.32-8.10) |  |  |
|  | 30→40 | Stable obesity | 0.58 (0.13-2.50) | 0.10 (0.01-0.94) |  |  |
|  | 40→Current | Maximum overweight | 1.98 (0.96-4.10) | 2.01 (0.66-6.10) | 0.572 | 0.801 |
|  | 40→Current | Non-obese → obese | 1.42 (0.45-4.50) | 0.89 (0.20-3.93) |  |  |
|  | 40→Current | Obese → non-obese | 2.03 (0.41-10.00) | NA |  |  |
|  | 40→Current | Stable obesity | 1.20 (0.36-4.00) | 0.96 (0.21-4.43) |  |  |
|  | 18→Current | Maximum overweight | 1.93 (0.96-3.88) | 2.02 (0.71-5.76) | 0.257 | 0.980 |
|  | 18→Current | Non-obese → obese | 1.31 (0.51-3.38) | 1.18 (0.34-4.12) |  |  |
|  | 18→Current | Obese → non-obese | 2.76 (0.41-18.43) | 0.23 (0.00-37.04) |  |  |
|  | 18→Current | Stable obesity | 0.14 (0.00-4.03) | NA |  |  |

Abbreviations: CAP, controlled attenuation parameter; OR, odds ratio; CI, confidence interval.

Trajectory definition: BMI was categorized at each time point using Chinese cut-offs (<24.0, 24.0–27.9, and ≥28.0 kg/m²). Trajectory patterns were defined for each interval (18→30, 30→40, 40→current, and 18→current) and included maximum overweight, non-obese→obese, obese→non-obese, and stable obesity; stable normal weight served as the reference group.

Models were adjusted for age, sex, ethnicity, smoking status, past-year alcohol drinking frequency, waist circumference, and metabolic/liver biomarkers (ALT, AST, ALP, HDL-C, LDL-C, triglycerides, total cholesterol, fasting blood glucose, and uric acid). The stratification variable was not additionally adjusted within that subgroup analysis.

P for interaction: P for interaction was obtained by testing the trajectory × subgroup interaction terms in Model 2 (Wald test) using the full analytic sample; P values are shown separately for CAP ≥238 dB/m and CAP ≥292 dB/m.

**Supplementary Table S3. Subgroup analyses of BMI trajectory patterns in relation to CAP-defined steatosis and severe steatosis by age**

| **Sex level** | **Interval** | **Trajectory pattern (ref: Stable normal)** | **CAP ≥238 OR (95% CI)** | **CAP ≥292 OR (95% CI)** | **P for interaction (CAP ≥238)** | **P for interaction (CAP ≥292)** |
| --- | --- | --- | --- | --- | --- | --- |
| <50 | 18→30 | Maximum overweight | 0.97 (0.47-2.00) | 0.92 (0.40-2.14) | 0.647 | 0.786 |
|  | 18→30 | Non-obese → obese | 1.12 (0.27-4.71) | 1.09 (0.30-3.93) |  |  |
|  | 18→30 | Obese → non-obese | NA | 1.79 (0.10-32.38) |  |  |
|  | 18→30 | Stable obesity | 0.01 (0.00-28.54) | NA |  |  |
|  | 30→40 | Maximum overweight | 1.45 (0.70-3.03) | 0.65 (0.26-1.60) | 0.745 | 0.984 |
|  | 30→40 | Non-obese → obese | 0.91 (0.25-3.26) | 0.67 (0.19-2.38) |  |  |
|  | 30→40 | Obese → non-obese | 1.03 (0.15-7.20) | 1.46 (0.23-9.09) |  |  |
|  | 30→40 | Stable obesity | 0.88 (0.16-4.93) | 0.42 (0.08-2.27) |  |  |
|  | 40→Current | Maximum overweight | 2.35 (1.01-5.45) | 6.52 (0.82-51.80) | 0.296 | 0.727 |
|  | 40→Current | Non-obese → obese | 3.54 (0.75-16.70) | 3.19 (0.32-31.43) |  |  |
|  | 40→Current | Obese → non-obese | 1.01 (0.23-4.42) | 2.28 (0.17-30.40) |  |  |
|  | 40→Current | Stable obesity | 2.50 (0.48-12.94) | 4.18 (0.41-43.04) |  |  |
|  | 18→Current | Maximum overweight | 1.59 (0.74-3.41) | 4.55 (0.98-21.10) | 0.931 | 0.813 |
|  | 18→Current | Non-obese → obese | 2.70 (0.76-9.62) | 2.90 (0.53-16.00) |  |  |
|  | 18→Current | Obese → non-obese | NA | NA |  |  |
|  | 18→Current | Stable obesity | 0.83 (0.06-11.93) | 1.40 (0.09-21.72) |  |  |
| ≥50 | 18→30 | Maximum overweight | 1.62 (0.89-2.94) | 1.52 (0.76-3.05) | 0.647 | 0.786 |
|  | 18→30 | Non-obese → obese | 0.93 (0.29-3.02) | 2.23 (0.74-6.73) |  |  |
|  | 18→30 | Obese → non-obese | 1.13 (0.20-6.36) | 0.57 (0.04-8.68) |  |  |
|  | 18→30 | Stable obesity | 1.30 (0.12-14.12) | 0.44 (0.04-4.41) |  |  |
|  | 30→40 | Maximum overweight | 1.19 (0.66-2.13) | 0.83 (0.40-1.73) | 0.745 | 0.984 |
|  | 30→40 | Non-obese → obese | 2.40 (0.79-7.26) | 0.98 (0.33-2.90) |  |  |
|  | 30→40 | Obese → non-obese | 0.87 (0.23-3.22) | 2.17 (0.62-7.60) |  |  |
|  | 30→40 | Stable obesity | 1.24 (0.22-7.17) | 0.59 (0.12-2.93) |  |  |
|  | 40→Current | Maximum overweight | 1.65 (0.88-3.09) | 1.61 (0.66-3.95) | 0.296 | 0.727 |
|  | 40→Current | Non-obese → obese | 2.02 (0.66-6.17) | 0.93 (0.27-3.28) |  |  |
|  | 40→Current | Obese → non-obese | 5.65 (1.40-22.86) | 0.31 (0.03-3.42) |  |  |
|  | 40→Current | Stable obesity | 1.68 (0.45-6.26) | 1.70 (0.46-6.38) |  |  |
|  | 18→Current | Maximum overweight | 1.95 (1.07-3.56) | 1.56 (0.66-3.70) | 0.931 | 0.813 |
|  | 18→Current | Non-obese → obese | 1.75 (0.68-4.52) | 1.32 (0.44-3.97) |  |  |
|  | 18→Current | Obese → non-obese | 1.24 (0.26-5.91) | 0.12 (0.00-13.40) |  |  |
|  | 18→Current | Stable obesity | NA | 0.85 (0.09-7.74) |  |  |

Abbreviations: CAP, controlled attenuation parameter; OR, odds ratio; CI, confidence interval.

Trajectory definition: BMI was categorized at each time point using Chinese cut-offs (<24.0, 24.0–27.9, and ≥28.0 kg/m²). Trajectory patterns were defined for each interval (18→30, 30→40, 40→current, and 18→current) and included maximum overweight, non-obese→obese, obese→non-obese, and stable obesity; stable normal weight served as the reference group.

Models were adjusted for age, sex, ethnicity, smoking status, past-year alcohol drinking frequency, waist circumference, and metabolic/liver biomarkers (ALT, AST, ALP, HDL-C, LDL-C, triglycerides, total cholesterol, fasting blood glucose, and uric acid). The stratification variable was not additionally adjusted within that subgroup analysis.

P for interaction: P for interaction was obtained by testing the trajectory × subgroup interaction terms in Model 2 (Wald test) using the full analytic sample; P values are shown separately for CAP ≥238 dB/m and CAP ≥292 dB/m.

**Supplementary Table S4. Subgroup analyses of BMI trajectory patterns in relation to CAP-defined steatosis and severe steatosis by age**

| **Sex level** | **Interval** | **Trajectory pattern (ref: Stable normal)** | **CAP ≥238 OR (95% CI)** | **CAP ≥292 OR (95% CI)** | **P for interaction (CAP ≥238)** | **P for interaction (CAP ≥292)** |
| --- | --- | --- | --- | --- | --- | --- |
| Han | 18→30 | Maximum overweight | 1.23 (0.47-3.20) | 0.93 (0.35-2.46) | 0.985 | 0.920 |
|  | 18→30 | Non-obese → obese | 1.44 (0.23-8.93) | 0.98 (0.18-5.25) |  |  |
|  | 18→30 | Obese → non-obese | NA | 0.92 (0.03-32.51) |  |  |
|  | 18→30 | Stable obesity | NA | 0.06 (0.00-4.22) |  |  |
|  | 30→40 | Maximum overweight | 0.87 (0.35-2.17) | 0.30 (0.11-0.85) | 0.256 | 0.254 |
|  | 30→40 | Non-obese → obese | 0.22 (0.04-1.32) | 0.83 (0.17-4.14) |  |  |
|  | 30→40 | Obese → non-obese | 1.11 (0.17-7.33) | 0.76 (0.12-4.96) |  |  |
|  | 30→40 | Stable obesity | NA | 0.12 (0.01-1.55) |  |  |
|  | 40→Current | Maximum overweight | 3.04 (1.18-7.83) | 1.62 (0.47-5.61) | 0.396 | 0.622 |
|  | 40→Current | Non-obese → obese | 2.76 (0.47-16.35) | 0.41 (0.07-2.27) |  |  |
|  | 40→Current | Obese → non-obese | 1.31 (0.10-16.87) | NA |  |  |
|  | 40→Current | Stable obesity | 0.74 (0.09-5.92) | 1.88 (0.27-13.14) |  |  |
|  | 18→Current | Maximum overweight | 4.01 (1.59-10.15) | 1.73 (0.52-5.81) | 0.205 | 0.935 |
|  | 18→Current | Non-obese → obese | 1.51 (0.35-6.53) | 1.06 (0.23-4.89) |  |  |
|  | 18→Current | Obese → non-obese | NA | NA |  |  |
|  | 18→Current | Stable obesity | NA | 0.34 (0.02-5.96) |  |  |
| Minority | 18→30 | Maximum overweight | 1.22 (0.72-2.08) | 1.30 (0.67-2.50) | 0.985 | 0.920 |
|  | 18→30 | Non-obese → obese | 0.75 (0.26-2.19) | 1.91 (0.71-5.13) |  |  |
|  | 18→30 | Obese → non-obese | 1.13 (0.20-6.41) | 0.97 (0.11-8.50) |  |  |
|  | 18→30 | Stable obesity | 0.41 (0.06-2.95) | 0.28 (0.02-4.78) |  |  |
|  | 30→40 | Maximum overweight | 1.30 (0.77-2.22) | 1.01 (0.50-2.05) | 0.256 | 0.254 |
|  | 30→40 | Non-obese → obese | 2.07 (0.80-5.36) | 0.82 (0.30-2.22) |  |  |
|  | 30→40 | Obese → non-obese | 0.66 (0.17-2.64) | 2.47 (0.69-8.82) |  |  |
|  | 30→40 | Stable obesity | 0.78 (0.21-2.81) | 0.68 (0.18-2.62) |  |  |
|  | 40→Current | Maximum overweight | 1.40 (0.77-2.56) | 2.16 (0.80-5.84) | 0.396 | 0.622 |
|  | 40→Current | Non-obese → obese | 2.00 (0.68-5.87) | 1.50 (0.41-5.43) |  |  |
|  | 40→Current | Obese → non-obese | 2.13 (0.71-6.38) | 0.85 (0.17-4.30) |  |  |
|  | 40→Current | Stable obesity | 1.94 (0.58-6.45) | 1.57 (0.41-6.07) |  |  |
|  | 18→Current | Maximum overweight | 1.21 (0.68-2.16) | 2.15 (0.87-5.34) | 0.205 | 0.935 |
|  | 18→Current | Non-obese → obese | 1.76 (0.70-4.38) | 1.67 (0.54-5.16) |  |  |
|  | 18→Current | Obese → non-obese | 0.96 (0.21-4.49) | NA |  |  |
|  | 18→Current | Stable obesity | 0.57 (0.05-6.88) | 1.40 (0.16-12.60) |  |  |

Abbreviations: CAP, controlled attenuation parameter; OR, odds ratio; CI, confidence interval.

Trajectory definition: BMI was categorized at each time point using Chinese cut-offs (<24.0, 24.0–27.9, and ≥28.0 kg/m²). Trajectory patterns were defined for each interval (18→30, 30→40, 40→current, and 18→current) and included maximum overweight, non-obese→obese, obese→non-obese, and stable obesity; stable normal weight served as the reference group.

Models were adjusted for age, sex, ethnicity, smoking status, past-year alcohol drinking frequency, waist circumference, and metabolic/liver biomarkers (ALT, AST, ALP, HDL-C, LDL-C, triglycerides, total cholesterol, fasting blood glucose, and uric acid). The stratification variable was not additionally adjusted within that subgroup analysis.

P for interaction: P for interaction was obtained by testing the trajectory × subgroup interaction terms in Model 2 (Wald test) using the full analytic sample; P values are shown separately for CAP ≥238 dB/m and CAP ≥292 dB/m.

**Supplementary Table S5. Subgroup analyses of BMI trajectory patterns in relation to CAP-defined steatosis and severe steatosis by smoking status**

| **Sex level** | **Interval** | **Trajectory pattern (ref: Stable normal)** | **CAP ≥238 OR (95% CI)** | **CAP ≥292 OR (95% CI)** | **P for interaction (CAP ≥238)** | **P for interaction (CAP ≥292)** |
| --- | --- | --- | --- | --- | --- | --- |
| No | 18→30 | Maximum overweight | 1.06 (0.59-1.91) | 0.95 (0.46-1.99) | 0.893 | 0.028 |
|  | 18→30 | Non-obese → obese | 0.95 (0.29-3.13) | 0.55 (0.17-1.80) |  |  |
|  | 18→30 | Obese → non-obese | 1.85 (0.18-18.88) | 0.68 (0.03-17.11) |  |  |
|  | 18→30 | Stable obesity | 0.77 (0.07-8.22) | 0.01 (0.00-0.73) |  |  |
|  | 30→40 | Maximum overweight | 0.96 (0.53-1.71) | 0.73 (0.34-1.57) | 0.514 | 0.063 |
|  | 30→40 | Non-obese → obese | 1.00 (0.33-2.97) | 0.61 (0.20-1.92) |  |  |
|  | 30→40 | Obese → non-obese | 1.31 (0.27-6.44) | 0.89 (0.21-3.80) |  |  |
|  | 30→40 | Stable obesity | 0.63 (0.15-2.67) | 0.08 (0.01-0.71) |  |  |
|  | 40→Current | Maximum overweight | 1.89 (0.99-3.61) | 1.77 (0.67-4.66) | 0.906 | 0.282 |
|  | 40→Current | Non-obese → obese | 1.86 (0.63-5.50) | 1.00 (0.28-3.61) |  |  |
|  | 40→Current | Obese → non-obese | 2.61 (0.58-11.72) | 0.24 (0.02-2.55) |  |  |
|  | 40→Current | Stable obesity | 1.14 (0.35-3.69) | 0.78 (0.19-3.19) |  |  |
|  | 18→Current | Maximum overweight | 1.88 (1.01-3.48) | 1.57 (0.64-3.86) | 0.382 | 0.366 |
|  | 18→Current | Non-obese → obese | 1.48 (0.60-3.68) | 1.11 (0.37-3.39) |  |  |
|  | 18→Current | Obese → non-obese | 2.86 (0.43-18.94) | 0.13 (0.00-43.79) |  |  |
|  | 18→Current | Stable obesity | 0.51 (0.04-6.56) | 0.11 (0.01-2.00) |  |  |
| Yes | 18→30 | Maximum overweight | 1.58 (0.74-3.38) | 1.55 (0.68-3.50) | 0.893 | 0.028 |
|  | 18→30 | Non-obese → obese | 0.82 (0.19-3.53) | 6.59 (1.68-25.89) |  |  |
|  | 18→30 | Obese → non-obese | 1.14 (0.13-10.13) | 1.32 (0.13-13.54) |  |  |
|  | 18→30 | Stable obesity | 0.38 (0.03-5.09) | 2.17 (0.15-30.72) |  |  |
|  | 30→40 | Maximum overweight | 1.65 (0.79-3.45) | 0.66 (0.27-1.61) | 0.514 | 0.063 |
|  | 30→40 | Non-obese → obese | 2.33 (0.61-8.84) | 0.94 (0.28-3.17) |  |  |
|  | 30→40 | Obese → non-obese | 0.51 (0.10-2.60) | 3.58 (0.79-16.27) |  |  |
|  | 30→40 | Stable obesity | 1.81 (0.16-20.72) | 4.47 (0.55-36.39) |  |  |
|  | 40→Current | Maximum overweight | 1.57 (0.71-3.49) | 2.56 (0.68-9.66) | 0.906 | 0.282 |
|  | 40→Current | Non-obese → obese | 3.94 (0.66-23.42) | 1.13 (0.20-6.33) |  |  |
|  | 40→Current | Obese → non-obese | 1.93 (0.48-7.74) | 1.25 (0.16-10.00) |  |  |
|  | 40→Current | Stable obesity | NA | 5.25 (0.86-32.18) |  |  |
|  | 18→Current | Maximum overweight | 1.47 (0.69-3.11) | 3.28 (0.89-12.13) | 0.382 | 0.366 |
|  | 18→Current | Non-obese → obese | 4.67 (0.84-25.99) | 2.58 (0.54-12.24) |  |  |
|  | 18→Current | Obese → non-obese | 0.31 (0.02-4.04) | NA |  |  |
|  | 18→Current | Stable obesity | NA | 4.80 (0.42-54.63) |  |  |

Abbreviations: CAP, controlled attenuation parameter; OR, odds ratio; CI, confidence interval.

Trajectory definition: BMI was categorized at each time point using Chinese cut-offs (<24.0, 24.0–27.9, and ≥28.0 kg/m²). Trajectory patterns were defined for each interval (18→30, 30→40, 40→current, and 18→current) and included maximum overweight, non-obese→obese, obese→non-obese, and stable obesity; stable normal weight served as the reference group.

Models were adjusted for age, sex, ethnicity, smoking status, past-year alcohol drinking frequency, waist circumference, and metabolic/liver biomarkers (ALT, AST, ALP, HDL-C, LDL-C, triglycerides, total cholesterol, fasting blood glucose, and uric acid). The stratification variable was not additionally adjusted within that subgroup analysis.

P for interaction: P for interaction was obtained by testing the trajectory × subgroup interaction terms in Model 2 (Wald test) using the full analytic sample; P values are shown separately for CAP ≥238 dB/m and CAP ≥292 dB/m.

**Supplementary Table S6. Multiplicative interaction between current BMI category and alcohol drinking frequency for CAP-defined steatosis (CAP ≥238 dB/m).**

| **Interaction term** | **OR (95% CI)** | ***P*** |
| --- | --- | --- |
| BMI 24-27.9 × Alcohol 1 (ref: BMI <24 & Alcohol 0) | 1.15 (0.42-3.14) | 0.788 |
| BMI ≥28 × Alcohol 1 (ref: BMI <24 & Alcohol 0) | 3.02 (0.65-14.01) | 0.158 |
| BMI 24-27.9 × Alcohol 2 (ref: BMI <24 & Alcohol 0) | 1.03 (0.41-2.61) | 0.943 |
| BMI ≥28 × Alcohol 2 (ref: BMI <24 & Alcohol 0) | 5.27 (1.00-27.73) | 0.050 |
| BMI 24-27.9 × Alcohol 3 (ref: BMI <24 & Alcohol 0) | 0.68 (0.18-2.52) | 0.560 |
| BMI ≥28 × Alcohol 3 (ref: BMI <24 & Alcohol 0) | 1.13 (0.17-7.34) | 0.900 |
| BMI 24-27.9 × Alcohol ≥4 (ref: BMI <24 & Alcohol 0) | 0.75 (0.33-1.72) | 0.492 |
| BMI ≥28 × Alcohol ≥4 (ref: BMI <24 & Alcohol 0) | 1.43 (0.34-6.02) | 0.622 |

Overall interaction P (likelihood ratio test comparing models with vs without interaction terms): 0.503. OR (95% CI) are for interaction terms relative to the joint reference (BMI <24 and alcohol 0).

**Supplementary Table S7. Elevated liver stiffness (LSM ≥8.0 kPa) as a supplementary fibrosis-related outcome**

| **Section** | **Group** | **Elevated LSM, n/N (%)** | **Adjusted OR (95% CI)** | ***P* value** |
| --- | --- | --- | --- | --- |
| **Overall prevalence** | Overall primary analytic sample | 61/956 (6.4) | — | — |
| CAP-defined steatosis severity | CAP <238 dB/m | 21/450 (4.7) | Reference | — |
|  | CAP 238–258 dB/m | 6/141 (4.3) | 1.07 (0.41–2.78) | 0.897 |
|  | CAP 259–291 dB/m | 15/191 (7.9) | 2.01 (0.98–4.13) | 0.056 |
|  | CAP ≥292 dB/m | 19/174 (10.9) | 3.48 (1.72–7.02) | <0.001 |
| Current BMI category | BMI <24.0 kg/m² | 25/413 (6.1) | Reference | — |
|  | BMI 24.0–27.9 kg/m² | 23/354 (6.5) | 1.30 (0.70–2.39) | 0.403 |
|  | BMI ≥28.0 kg/m² | 13/189 (6.9) | 1.80 (0.86–3.77) | 0.117 |

**Notes.** Elevated liver stiffness was defined as LSM ≥8.0 kPa. Adjusted odds ratios are from core-adjusted logistic regression models controlling for age, sex, ethnicity, smoking status, and past-year alcohol drinking frequency. Trajectory-specific rows are presented descriptively because the number of elevated-LSM events was limited in several rare categories. Abbreviations: LSM, liver stiffness measurement; CAP, controlled attenuation parameter; BMI, body mass index; OR, odds ratio; CI, confidence interval.
